# Ultrahigh Adhesion Force Between Silica-Binding Peptide SB7 and Glass Substrate Studied by Single-Molecule Force Spectroscopy and Molecular Dynamic Simulation

**DOI:** 10.3389/fchem.2020.600918

**Published:** 2020-11-27

**Authors:** Xiaoxu Zhang, Jialin Chen, Enci Li, Chunguang Hu, Shi-Zhong Luo, Chengzhi He

**Affiliations:** ^1^Beijing Advanced Innovation Center for Soft Matter Science and Engineering, Beijing University of Chemical Technology, Beijng, China; ^2^Beijing Key Laboratory of Bioprocess, College of Life Science and Technology, Beijing University of Chemical Technology, Beijing, China; ^3^State Key Laboratory of Precision Measuring Technology and Instrument, Tianjin University, Tianjin, China

**Keywords:** adhesion, peptide, silica, single molecule force spectroscopy (SMFS), atomic force micorscopy (AFM), molecular dynamics simulation (MD)

## Abstract

Many proteins and peptides have been identified to effectively and specifically bind on certain surfaces such as silica, polystyrene and titanium dioxide. It is of great interest, in many areas such as enzyme immobilization, surface functionalization and nanotechnology, to understand how these proteins/peptides bind to solid surfaces. Here we use single-molecule force spectroscopy (SMFS) based on atomic force microscopy to directly measure the adhesion force between a silica-binding peptide SB7 and glass surface at single molecule level. SMFS results show that the adhesion force of a single SB7 detaching from the glass surface distributes in two populations at ~220 pN and 610 pN, which is higher than the unfolding forces of most mechanically stable proteins and the unbinding forces of most stable protein-protein interactions. Molecular dynamics simulation reveals that the electrostatic interactions between positively charged arginine residues and the silica surface dominates the binding of SB7 on silica. Our study provides experimental evidence and molecular mechanism at the single-molecule level for the SB7-based immobilization of proteins on silica-based surface, which is able to withstand high mechanical forces, making it an ideal fusion tag for silica surface immobilization or peptide-base adhesive materials.

## Introduction

Solid-binding peptides/proteins have been of great interest in many applications such as surface functionalization, fusion tags for protein purification, fabrication of functional architectures, drug delivery, and biological immobilization of enzymes (Ikeda et al., 2009, 2010; Coyle and Baneyx, 2014; Kim et al., 2019; Hellner et al., 2020). Silica is one of the most abundant materials in nature, widely used in structure materials, food industry, pharmaceutical applications, and semiconductors. A number of silica binding proteins/peptides have been found and designed (Table 1). The ribosomal protein L2, which is the 50S large ribosomal subunit, can bind strongly to the surface of silica and has been used as a fusion tag (Si-Tag) for surface functionalization and protein purification (Ikeda et al., 2009, 2010, 2011). Sunna et al. demonstrated that a recombinant Linker-Protein G (LPG) sequence has affinity to silica-containing materials (Sunna et al., 2013). Soto-Rodríguez et al. used Car9 as a fusion tag for proteins immobilization on silica matrices for the development of a rapid and inexpensive method of protein purification (Soto-Rodríguez et al., 2017).

**Table 1:** Silica-binding protein/peptides.

**Silica-binding protein/peptide**	**Sequence**
Si-Tag(Taniguchi et al., 2007; Ikeda et al., 2009)	MAVVKCKPTSPGRRHVVKVVNPELHKGKP FAPLLEKNSKSGGRNNNGRITTRHIGGGHK QVLGKAGAARWRGVRPTVRGTAMNPV DHPHGGGEGRNFGKHPVTPWGVQTKG KKTRSNKRTDKFIVRRRSK
Linker-Protein G (LPG)(Sunna et al., 2013)	(VKTQATSREEPPRLPSKHRPG)4VKTQTAS
Ect P1(Kim et al., 2019)	SSRSSSHRRHDHHDHRRGS
Car9(Coyle and Baneyx, 2014; Yang et al., 2016; Hellner et al., 2020)	DSARGFKKPGKR
CotB1p(Abdelhamid et al., 2014)	SGRARAQRQSSRGR
SB7(Abdelhamid et al., 2016)	RQSSRGR

It is important to understand the adhesion mechanism to find and design peptides/proteins with various binding strengths for different applications. Traditional methods such as surface plasma resonance (SPR) and quartz crystal microbalance (QCM) have been used to study the binding strength and molecular mechanism of the adhesion of peptides and proteins on different substrates (Hellner et al., 2020). Atomic force microscope (AFM) based single-molecule force spectroscopy (SMFS) has been widely used as a powerful tool to study the protein dynamics (Ott et al., 2017b), nano-mechanics of polymers (Cai et al., 2019; Song et al., 2019; Bao et al., 2020), the mechanical strength of single chemical bonds (Xue et al., 2014; Huang et al., 2019), the interaction of ligand-receptor (Lei et al., 2017a; Ott et al., 2017b; Li and Zheng, 2018) and single-molecule adhesions (Li et al., 2014, 2018, 2020; Maity et al., 2015; Pyles et al., 2019) with high force resolution. SMFS provides detailed information about peptides-surface interactions such as singe-molecule adhesion force that is not available with other techniques (Li et al., 2014, 2018, 2020; Maity et al., 2015). The peptide-surface interactions can be complex because of the existence of various types of interactions such as Van der Waals force, hydrogen bond, electrostatic interactions, hydrophobic effect, etc. To understand the key factor of in a peptide-silica adhesion, we aim to study the adhesion of peptide in a relatively simple system. Recently, a short peptide SB7 originated from the polycationic C-terminal sequence of spore coat protein *Bacillus cereus* CotB1 (171 amino acids) has been shown to bind the silica surface and used as an effectively affinity tag for protein purification (Abdelhamid et al., 2016). Such a short and simple sequence makes SB7 an ideal candidate for the study of adhesion mechanism.

**Figure 1:**
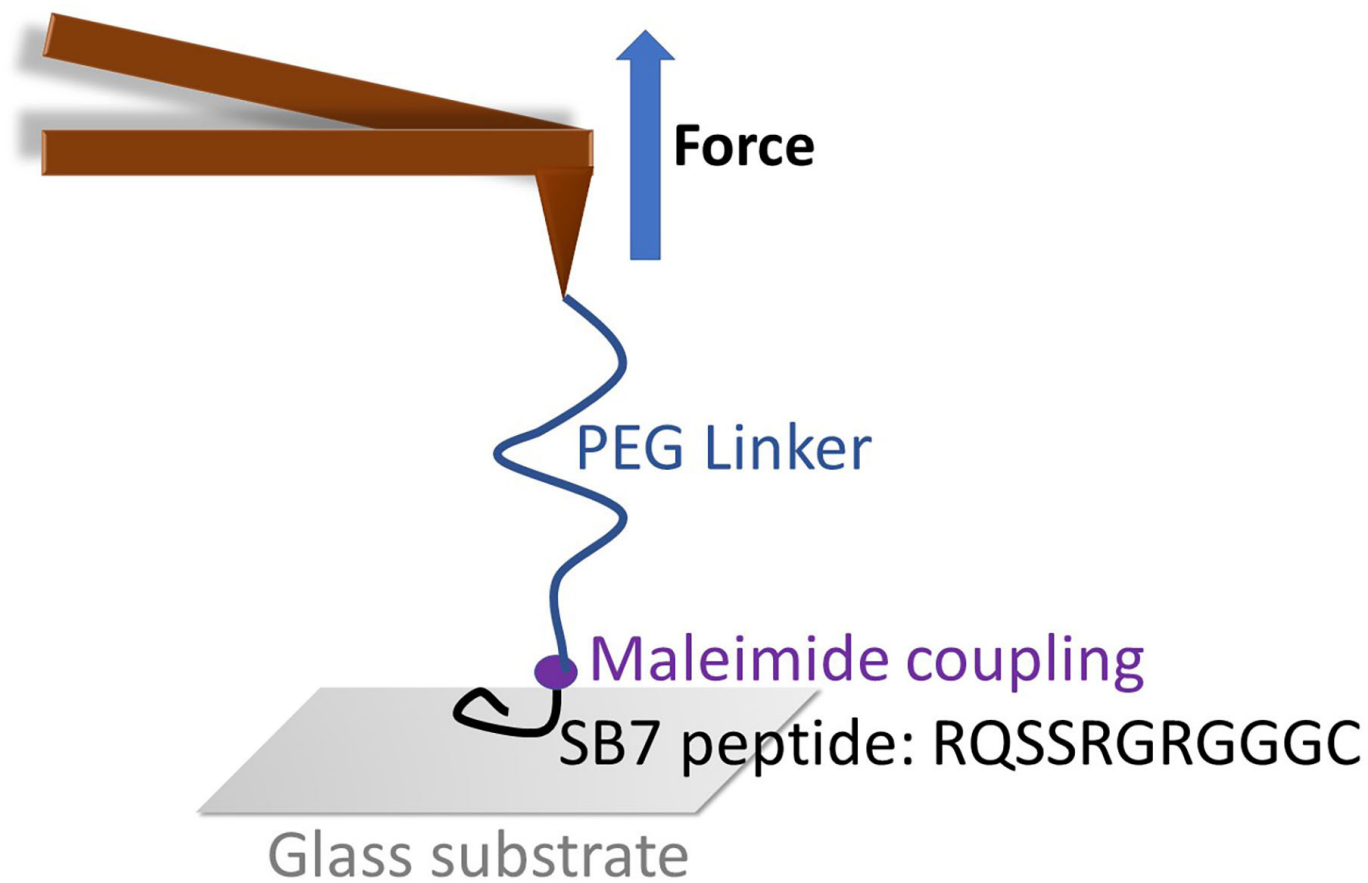
The schematic of SMFS experiment on SB7. The maleimide functionalized AFM tip was coupled with SB7 peptide through maleimide-thiol chemistry using a 35 kDa PEG linker.

In this work, we develop an AFM-based single-molecule force spectroscopy assay to quantitatively measure the adhesion force of SB7 on silica surface. The interactions of an AFM probe and substrate can be complex in a SMFS experiment. To minimize the influence of non-specific interactions often seen in SMFS experiments between the AFM tip and the substrate, we utilize a long Poly (ethylene-Glycol) linker to facilitate the identification of single-molecule adhesion force of peptide on silica substrate (Figure 1). In addition, molecular dynamics (MD) simulation was used to mimic the SMFS experiment and reveal the molecular mechanism of adhesion. For comparison, two additional silica-binding peptides, Car9 (DSARGFKKPGKR), and Arg7 (RRRRRRR) were also investigated using similar methods.

## Materials and Methods

### AFM Tip Functionalization and Glass Substrate Preparation

Poly (ethylene-Glycol) (molecular weight of ~35 kDa) with N-Hydroxysuccinimide (NHS) group at one end and a Maleimide (MAL) group at the other end (NHS-PEG-MAL) was purchased from Yarebio Inc., Shanghai. Poly (ethylene-Glycol) (molecular weight of ~20 kDa) with Hydroxysuccinimide (NHS) group at both ends (NHS-PEG-NHS) was purchased from Yarebio. The SB7 (RQSSRGRGGGC) and Car9 (DSARGFKKPGKRGGGC) peptides were synthesized by GenScript, Nanjing. The Arg7 (RRRRRRR) peptide was purchased from Allpeptide, Hanzhou.

The Si_3_N_4_ AFM tip (SiNi, BudgetSensors) was treated with UV/Ozone for 30 min and aminated by 1% (3-aminopropyl)triethoxysilane (APTES, Sigma-Aldrich) solution in ethanol for 1 h at room temperature. In the SMFS experiments on SB7 and Car9, the aminated AFM tip was then allowed to react with 5 mM NHS-PEG-MAL in Dimethylformamide (DMF) for 1 h at room temperature. Finally, 1 mg/mL SB7 or Car9 peptide solution in DMF was used to functionalize the AFM tip by coupling the thiol group from the cysteine residue of SB7 or Car9 and the maleimide group on the AFM tip at room temperature for 2 h. In the SMFS experiments on Arg7, the aminated AFM tip was then allowed to react with 5 mM NHS-PEG-NHS in Dimethylformamide (DMF) for 1 h at room temperature. Then, 1 mg/mL ARG7 peptide solution in DMF was used to functionalize the AFM tip by coupling the N-terminal amine group of R7 and the NHS group on the AFM tip at room temperature for 2 h. The glass substrate was treated with piranha solution (3:1 mixture of sulfuric acid and 30% hydrogen peroxide) for 1 h and rinsed with deionized water.

### Single-Molecule Force Spectroscopy Experiments

A commercial AFM (JPK Nonwizard 4) was used to perform SMFS experiments. The peptide-functionalized AFM tip (SiNi, BudgetSensors) was immersed in phosphate-buffered saline (PBS, pH = 7.4) and the spring constant was calibrated (~0.06 N/m) using equipartition theorem (Hutter and Bechhoefer, 1993). In a typical SMFS experiment, we moved the AFM tip toward the glass substrate and keep them in contact at the force of 0.5 nN for 0.5 s. Then the tip was retracted from the substrate at a certain pulling speed.

### Circular Dichroism Spectroscopy (CD)

The SB7 peptide was dissolved in water to minimize the background noise. Data were collected between 180 and 250 nm at 25°C using a Jasco J-815 CD spectrometer. A 1 mm path length quartz cuvette containing 200 μl SB7 (80 uM) was used for CD analysis.

### Molecular Dynamics Simulations

Molecular dynamics simulations were carried out using GROMACS 2016.3 (Heinz et al., 2005; Van Der Spoel et al., 2005; Abraham et al., 2015) with the charmm36 force field (Huang et al., 2016). Silica structure and force field were obtained through charmm-gui (Heinz et al., 2013; Hsu et al., 2017) and peptides were constructed as random coils using Pymol (Vinet and Zhedanov, 2011). The peptide was placed 1.5 nm above the silica. The system was solvated in water box using TIP3P model (Mark and Nilsson, 2001). The pH was set to 7.0 and 150 mM NaCl was added to the system. Periodic boundary conditions are used in the simulation. The non-bond cutoff distance was set to 1.2 nm. All MD simulations were performed using isothermal isostatic ensemble system (NPT) with a temperature of 298.15 K and a pressure of 1 atmosphere. The peptide was allowed to equilibrate and spontaneously absorb on the silica surface for 100 ns and then vertically pulled away from silica surface on C-terminus at the speed of 1 nm/ns for 4 ns while the silica substrate was fixed. VMD was used to visualize the peptide-silica system and GROMACS was used to analyze the data.

## Results

### Adhesion Force Revealed by Single-Molecule Force Spectroscopy

To investigate the adhesion force of silica-binding peptide/proteins on the silica surface, we performed single-molecule force spectroscopy experiments using atomic force spectroscopy on a short peptide SB7 (7 amino acid residues). The AFM probe was functionalized with the maleimide terminated PEG linker with the molecular weight of ~35 kDa. We added three glycine residues and one cysteine residue on the C-terminus of SB7 for the purpose of attachment of the peptide onto the AFM probe using maleimide-thiol reaction as shown in Figure 1.

**Figure 2:**
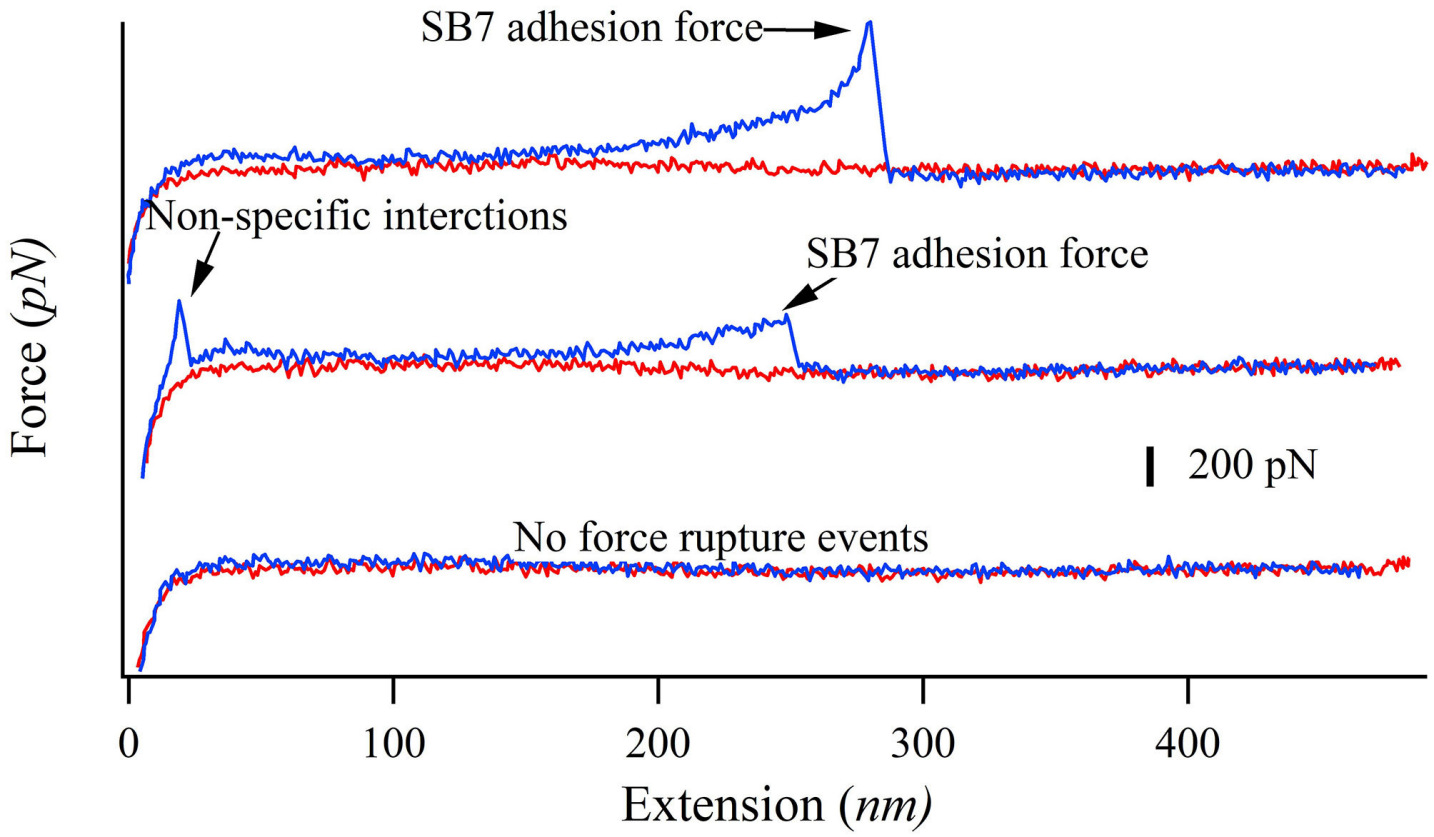
Representative force-extension curves of pulling SB7 from glass surface. The approaching and pulling curves are colored in red and blue, respectively. Top curve: single-molecule SB7 adhesion. Middle curve: single-molecule SB7 adhesion with non-specific interactions. Bottom curve: no force rupture events.

**Figure 3:**
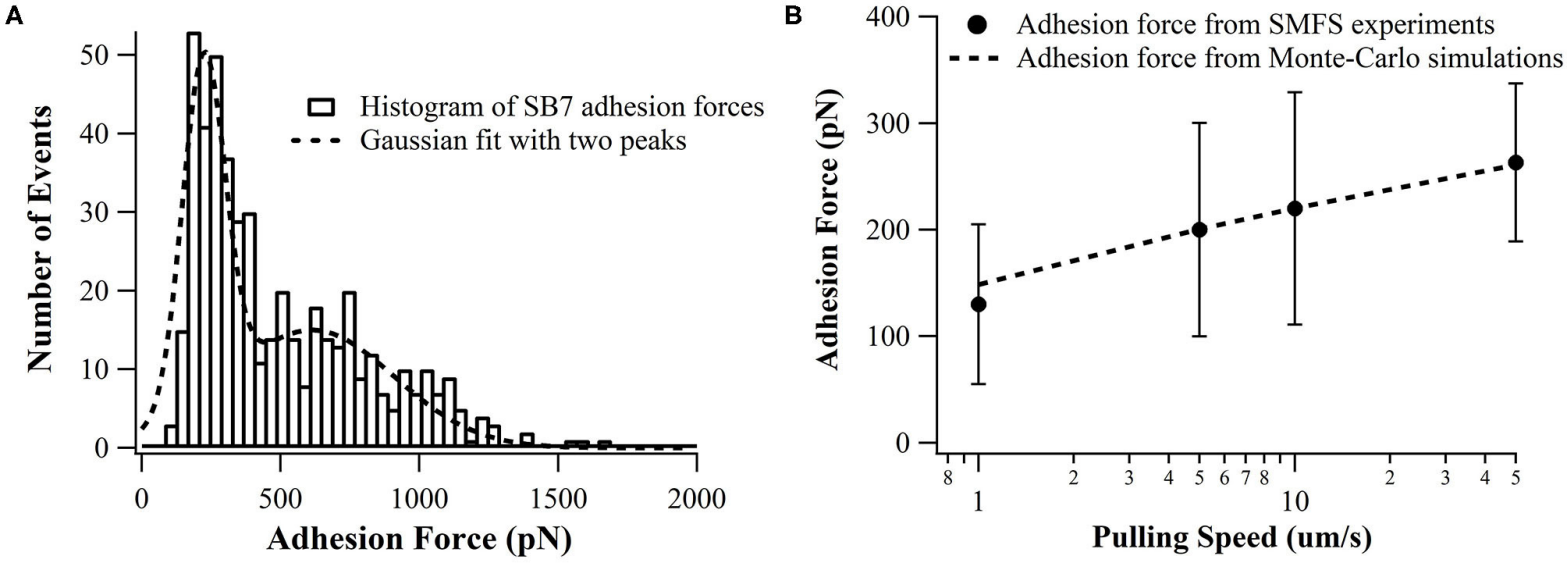
**(A)** Distributions adhesion force of SB7 on glass surface (pulling speed of 10 um/s). The histogram is fitted with two peaks using Gaussian model. The total number of events is 484. **(B)** The pulling speed dependence of adhesion force in SMFS experiments and Monte-Carlo simulations with the unbinding rate at zero force of 0.35 s^−1^ and unbinding distance of 0.16 nm using Bell-Evans model.

In our SMFS experiments, only about 5% of the pulling force-extension traces showed force rupture events as shown in Figure 2 (top and middle curves) while most pulling curves showed no force rupture events (bottom curve), which ensured the single-molecule pick-up. The rupture events at the extension of <100 nm often correspond to the non-specific interactions between the probe and the substrate as marked in Figure 2. The specific interactions between the SB7 and the glass surface can be identified at the extension of >200 nm due to the extension of the 35 kDa PEG linker. In this work, most of the 35 kDa PEG linker has a contour length of ~300 nm indicated by the worm-like-chain fitting using persistence length of 0.35 nm and contour length of 300 nm in Figure S1, which is in agreement with the theoretical calculation (~280 nm) as previously reported (Oesterhelt et al., 1999; Tong et al., 2013; Ott et al., 2017a). The distribution of the adhesion force between SB7 and silica substrate was showed in Figure 3. The histogram can be fitted to two peaks using Gaussian model. The main force peak centered at 220 pN has a smaller width of 109 pN while the minor force peak centered at 610 pN has larger width of 418 pN. This result indicates that there are two types of adhesion forces. To further characterize the kinetics of the unbinding of SB7 from glass surface, we performed SMFS experiments at different pulling velocities. By using the Monte-Carlo simulations and Bell-Evans model as previously reported (Bell, 1978; He et al., 2015; Lei et al., 2017b), we extracted the unbinding rate at zero force of 0.35 s^−1^ and unbinding distance (the distance from bonded state to transition state) of 0.16 nm (Figure 3B) for the main force peak at about 200 pN.

**Figure 4:**
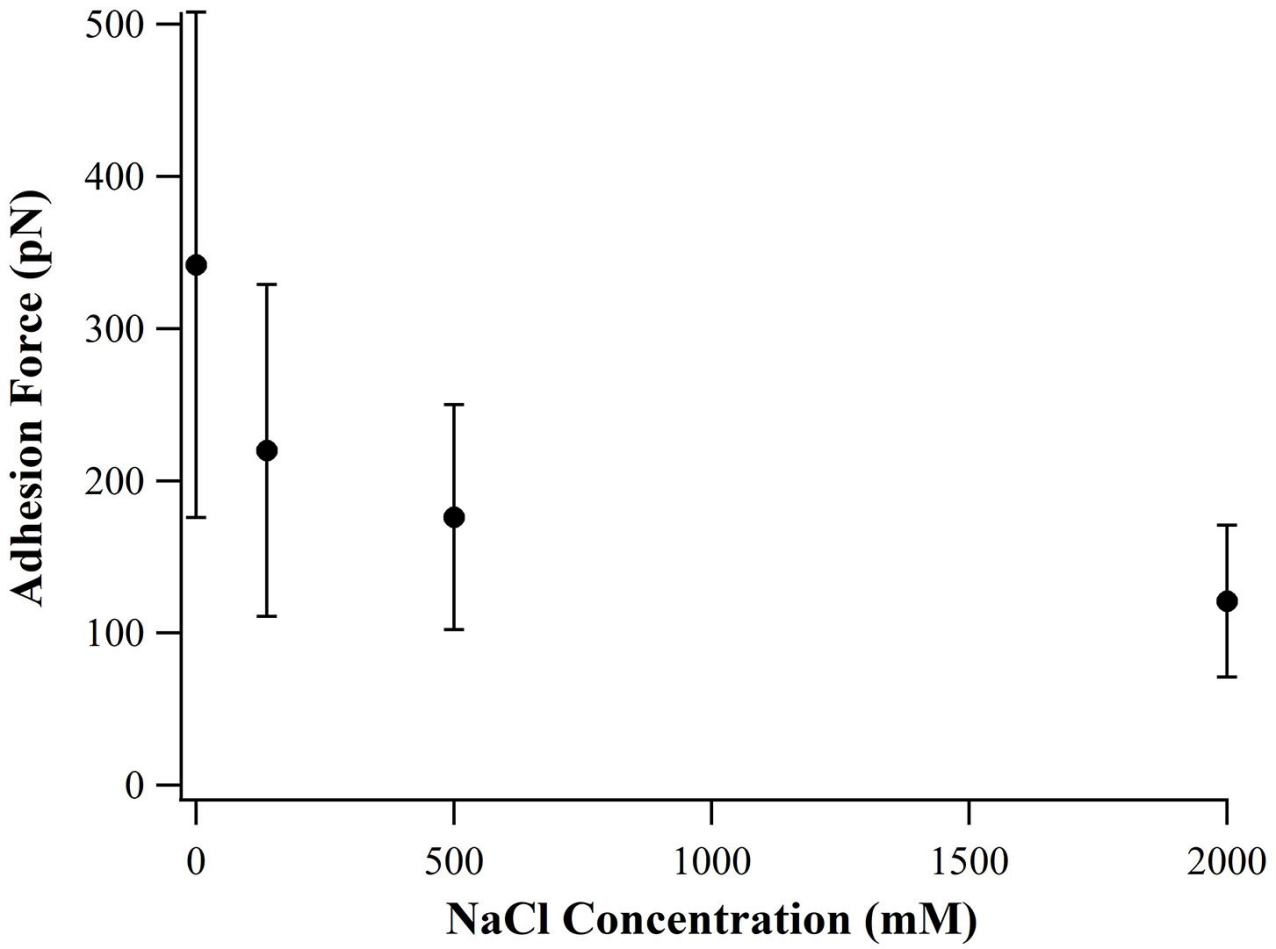
Adhesion force of SB7 at different concentration of NaCl (pulling speed of 10 um/s).

By examining the sequence of SB7, we suspect that the high adhesion force originated from electrostatic interactions between the positively charged arginine residues binding on the negatively charged silica surface. Higher ionic strength results in shorter Debye length and thus the electrostatic interactions between arginine residues and silica surface is weaker at higher ionic strength. By altering the ionic strength in SMFS experiments, we found that the adhesion forces decrease as the concentration of NaCl increases as shown in Figure 4, which supported the statement that the adhesion force originated from electrostatic interactions.

**Figure 5:**
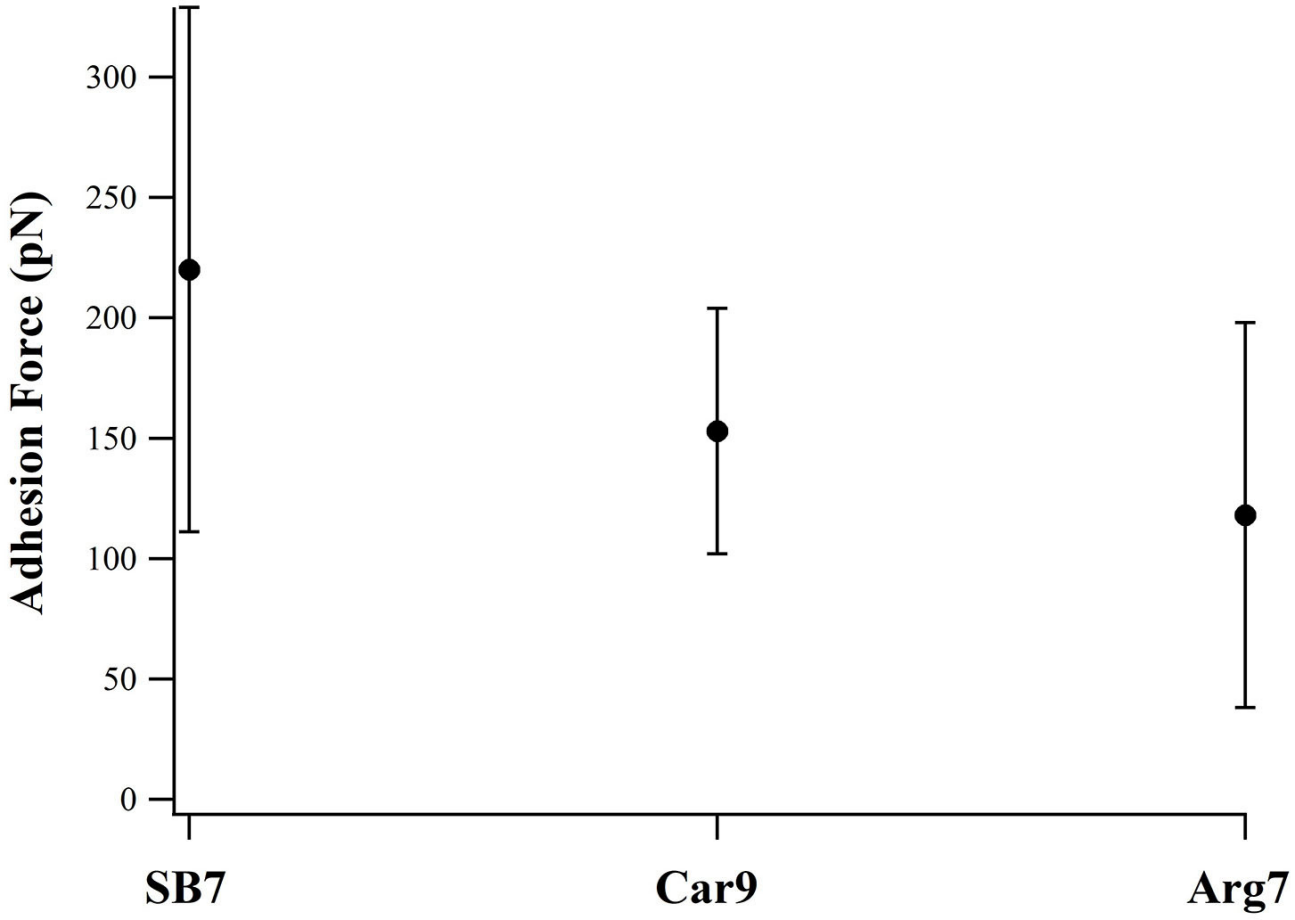
Adhesion forces of SB7, Car9, and Arg7 on glass surface (pulling speed of 10 um/s).

For comparison, we investigated two additional peptides Car9 and Arg7 using SMFS. There are five positively charged residues in Car9 (three lysine residues and two arginine residues). As showed in Figure 5, SB7 detached from the glass surface at the highest adhesion force among these three peptides. The result indicates that the lysine residues may has lower adhesion force than arginine residues when we compare the adhesion forces of SB7 and Car9. The lower adhesion force of Arg7 indicates the possibility that not all the arginine residues could bind tightly on the silica surface.

### Molecular Mechanism Revealed by Molecular Dynamics Simulation

**Figure 6:**
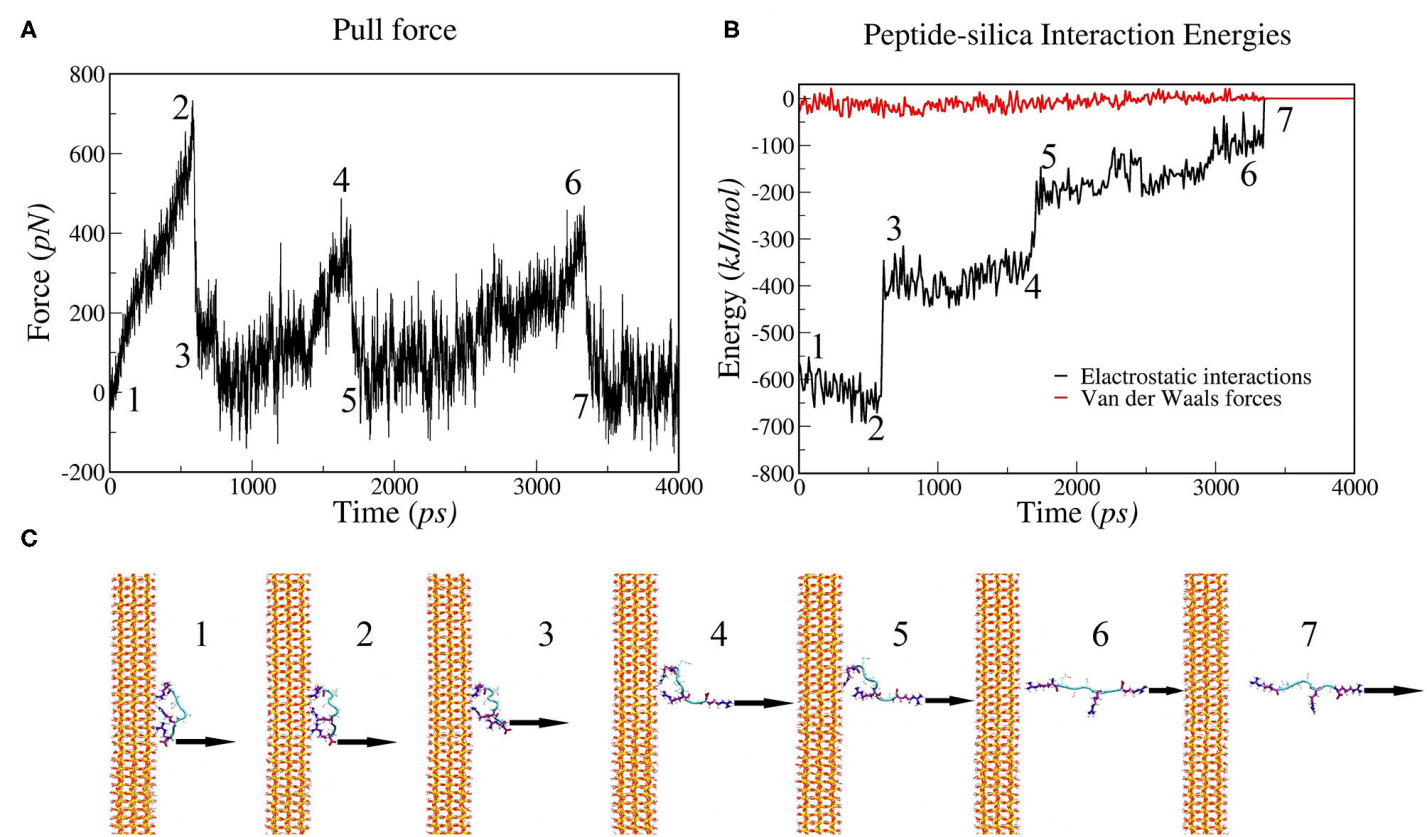
MD simulations of pulling SB7 (RQSSRGR) away from silica surface. **(A)** Force changes over time (constant pulling speed of 1 nm/ns); **(B)** Energy changes over time. **(C)** Snapshots of peptide-silica structure. The numbers labeled on **(A,B)** correspond the time of the snapshots in **(C)**. 1: The structure before pulling; 2 and 3: the structure before and after the R7 residue unbinding, respectively; 4 and 5: the structure before and after the R5 residue unbinding; 6 and 7: the structure before and after the R1 residue unbinding.

To investigate the molecular mechanism of the unbinding of SB7 peptide from the silica surface, we conducted all-atom MD simulations using GROMACS to mimic our SMFS experiments. The SB7 peptide was placed 1.5 nm above the silica surface, allowed to equilibrate for 100 ns and then pulled at 1 nm/ns. The distances between SB7 and silica surface before and after the equilibration for 100 ns are 1.5 and 0.9 nm, respectively. As shown in Figure 6, the SB7 peptide completely detached from the silica surface within 4 ns. As SB7 being pulled away, the pulling force, electrostatic interactions and Van der Waals force all dropped to zero. Three force peaks in Figure 6A and three electrostatic interactions steps in Figure 6B correspond to the unbinding of three positively charged arginine residues (R7, R5, and R1) from the silica surface as indicated in the snapshots in Figure 6C. Energy analysis revealed that the electrostatic interactions between the peptide and the silica surface are the main energy barriers during the unbinding process while the Van der Waals forces are relatively weak. Among the three arginine residues, R7 has the highest adhesion force upon being pulled from silica surface. In 10 repeats of MD simulations, R7, R5, and R1 unbind from the silica surface at average forces of 720, 342 and 377 pN, respectively (Figure S2). Pulling on N-terminal of SB7 generated similar results that the sequential unbinding of R1, R5, and R7 dominates the unbinding forces (Figure S3). To further study the molecular mechanism of peptide adhesion on silica surface, we performed MD simulations on Car9 and Arg7 peptides using the same methods. Our results indicate that the unbinding force of lysine residue in Car9 was much lower than the unbinding force of arginine residue (Figure S4). This is possibly because arginine residue has stronger electrostatic interaction with silica surface.

## Discussion

The surface layer of silica can be hydrolyzed to form silanol group (Si-OH) in aqueous solution and negatively charged when pH > 7, making it possible for the positively charged proteins/peptides to bind on silica surfaces through electrostatic interaction. There are different mechanism of adhesion forces of peptides/proteins on solid surface according to recent studies. The adhesion force of class II hydrophobin, a protein containing ~100 amino acid residues, on polystyrene surface has been characterized to be ~ 110 pN, mainly due to the hydrophobic effect (Li et al., 2018). A short peptide QPASSRY was found to bind on mica with an adhesion force ~100 pN, where the conformational freedom of the peptide contributes to the binding on the surface (Maity et al., 2015). Circular dichroism spectroscopy confirmed that the secondary structure of SB7 was random coils (Figure S5), indicating that the flexibility of the peptide chain may contribute to the binding on silica. *De novo* designed helical proteins have been shown to bind tightly on mica surface by utilizing the electrostatic interactions between negatively charged amino acid residues from proteins and cations on the mica surface (Pyles et al., 2019). Recently, a SMFS study on the dipeptide of lysine (Lys, K) and 3,4-Dihydroxyphenylalanine (DOPA, Y) residue, KY, also showed two distributions of adhesion force on TiO_2_ at ~100 pN and ~ 600 pN, which is similar to our results of adhesion force of SB7 on silica. Substitution of DOPA with phenylalanine showed no detectable adhesion force on TiO_2_, suggesting that DOPA is responsible for the binding on TiO_2_ (Li et al., 2020).

In this work, we reveal an arginine-based electrostatic interactions mechanism of adhesion for a short peptide SB7 binding on silica with ultrahigh adhesion force that exceeds the mechanical stability of most proteins and protein-ligand interactions. Our SMFS experiments showed two types of adhesion forces (~220 pN and ~600 pN). There are two possible explanations. On one hand, it is possible that there are two different binding modes/conformations for SB7 on the silica surface, which lead to the difference in adhesion forces. In 10 repeats of MD simulations, the SB7 absorbed on silica surface with the three arginine residues tightly bind on the surface and generated three individual adhesion force peaks. Among the three arginine residues, R7 has higher adhesion force than R5 and R1. In the SMFS experiments, only one force peak was observed for SB7 adhesion force. As the distances between R7, R5, and R1 are extremely small [(Wright et al., 2003)~1 nm], the long PEG linkers we used in SMFS affect the shape of the force-extension curves and may prevent the observation of the individual force peaks in SMFS experiments (Lyu et al., 2018). It was also possible that these arginine residues may unbind from the silica surface cooperatively or one or more arginine residues were not bond to the silica surface in the case of lower adhesion forces measured in SMFS experiments. In addition, the SMFS experiments may have different pulling direction from the perpendicular direction in the MD simulations. On the other hand, pulling on the same binding conformation could experience two different unbinding pathways, which has been widely observed in protein folding/unfolding dynamics (Wright et al., 2003; He et al., 2012, 2014). However, this is not observed in our simulation as all the simulations showed similar unbinding pathway.

Another silica binding peptide, Car9 (DSARGFKKPGKR) (Coyle and Baneyx, 2014) can bind to the silanol-rich surface of silica. By using surface plasma resonance (SPR), the equilibrium dissociation constant of Car9 on silica beads was determined to be ~1 uM (Coyle and Baneyx, 2014). Hellner et al. studied the binding mechanism of Car9 on silica surface using both SPR and MD simulations (Hellner et al., 2020). Both the lysine and arginine residues are important for Car9 to bind on silica. The glass binding assay using SPR indicated that Car9 binds strongly on glass surface and mutants of K8A-K11A and R4Q-R12Q showed significantly reduced binding ability to silica compared to wild-type Car9. In addition, the R4, K7, and K8 residues bind to silica tightly as the primary anchor residues and the K11 and R12 showed more variability as secondary anchor residues (Hellner et al., 2020). Our results from SMFS and MD simulations on Car9 indicate that arginine bind to the silica surface more tightly than lysine does. Compared to Car9, SB7 has no lysine residues and its sequence is shorter than Car9, making it possibly bind to silica through a simpler mechanism. However, the Arg7 (RRRRRRR) was reported to have a much lower binding affinity on silica than SB7, suggesting the importance of the non-charged residues as spacers in SB7 on the silica binding (Abdelhamid et al., 2016). Indeed, our SMFS experiments showed that the adhesion force of the Arg7 was lower than both SB7 and Car9, which suggest that the adhesion behavior of peptide on solid surface can be very complex.

## Conclusions

Our results indicate that SB7 binds strongly on silica surface at single-molecule level. The long PEG linker provides a simple yet effective way to distinguish specific adhesion events from non-specific interactions. Our MD simulations suggest the electrostatic interactions between the arginine residues and silica surface dominate the adhesion. Compared to other adhesive protein/peptides, SB7 has short sequence and ultrahigh adhesion force on its binding surface, making it an ideal fusion tag for proteins that need to be immobilized on silica-base materials.

## Data Availability Statement

The raw data supporting the conclusions of this article will be made available by the authors, without undue reservation.

## Author Contributions

XZ performed the SMFS experiments and analyzed the SMFS data. JC performed the MD simulations and analyzed the data. EL analyzed the SMFS data. CHu and S-ZL revised the manuscript. CHe designed this project, analyzed the data, and wrote the manuscript. All authors contributed to the article and approved the submitted version.

## Conflict of Interest

The authors declare that the research was conducted in the absence of any commercial or financial relationships that could be construed as a potential conflict of interest.
