# Supplementary material for: Ultrahigh Adhesion Force Between Silica-Binding Peptide SB7 and Glass Substrate Studied by Single-Molecule Force Spectroscopy and Molecular Dynamic Simulation
Source: Front Chem. 2020 Nov 27;8:600918. doi: 10.3389/fchem.2020.600918 (PMC7729015; doi:10.3389/fchem.2020.600918)
Supplement: Supplementary file 1 [file Table_1.DOCX]

Ultrahigh Adhesion Force between Silica-Binding Peptide SB7 and Glass Substrate Studied by Single-Molecule Force Spectroscopy and Molecular Dynamics Simulation

Xiaoxu Zhang^1#^, Jialin Chen^2#^, Enci Li^3^, Chunguang Hu^3^, Shi-zhong Luo^2^ and Chengzhi He^1*^

^1^ Beijing Advanced Innovation Center for Soft Matter Science and Enginering, Beijing University of Chemical Technology, 15 Beisanhuan Donglu, Beijng, 100029, China
^2^ Beijing Key Laboratory of Bioprocess, College of Life Science and Technology, Beijing University of Chemical Technology, 15 Beisanhuan Donglu, Beijing, 100029, China
^3^ State Key Laboratory of Precision Measuring Technology and Instrument, Tianjin University, 92 Weijin Rd, Tianjin, 300072, China

#These authors contribute to this work equally.

***Correspondence:**Chengzhi He
[czhe@mail.buct.edu.cn](mailto:czhe@mail.buct.edu.cn)

Keywords: Adhesion, Peptide, Silica, Single-Molecule Force Spectroscopy, Atomic Force Microscopy, Molecular Dynamics Simulation.

Supplementary Material

# Supplementary Figures and Tables

## Supplementary Figures


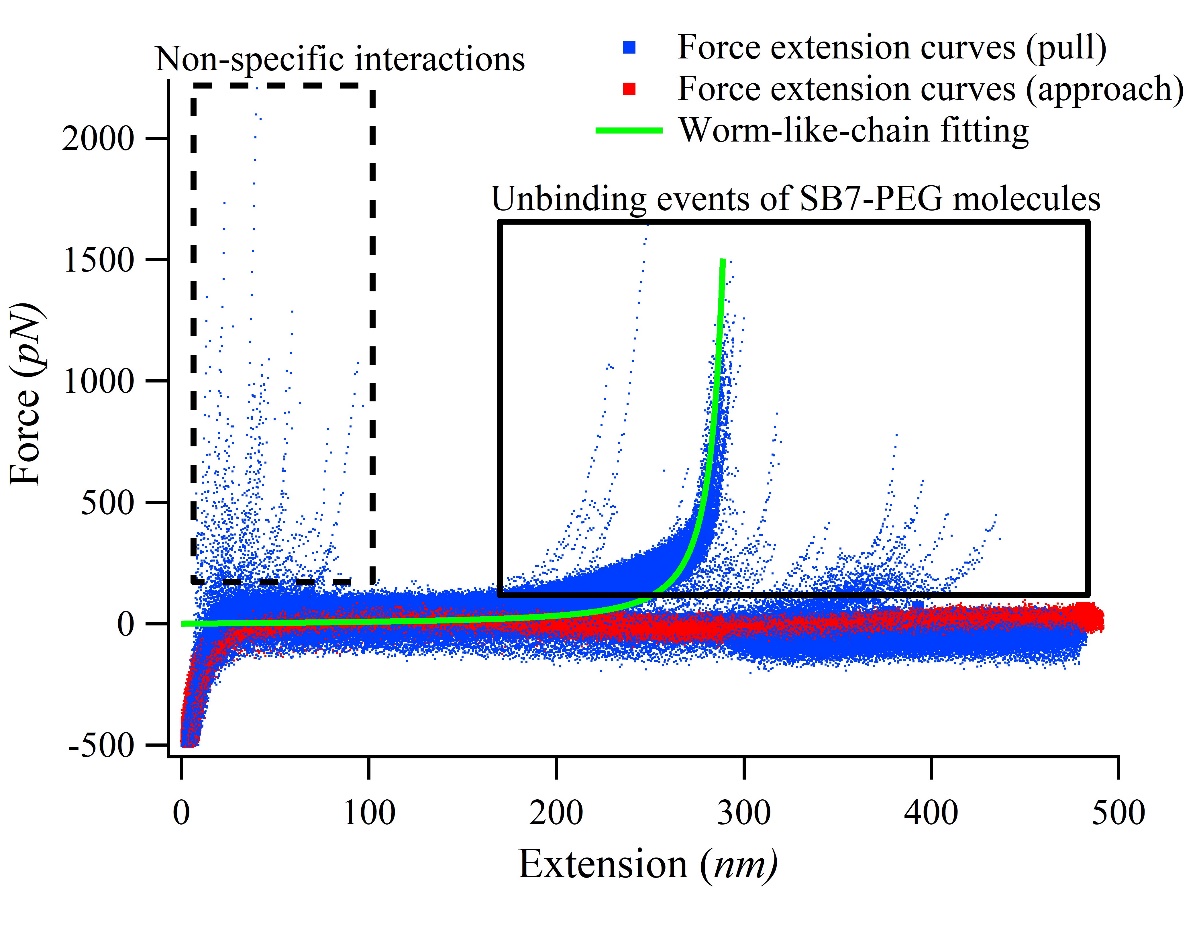


**Figure S1.** Force extension curves of pulling SB7 from glass surface with worm-like-chain fitting. 482 curves are overlapped. The pulling and approaching curves are colored in blue and red, respectively. The non-specific interactions are boxed with dashed black line at the extension of <100 nm. The unbinding events of SB7-PEG molecules are boxed with solid black line. Worm-like-chain fitting with persistence length of 0.35 nm and contour length of 300 nm is drew using solid green line.


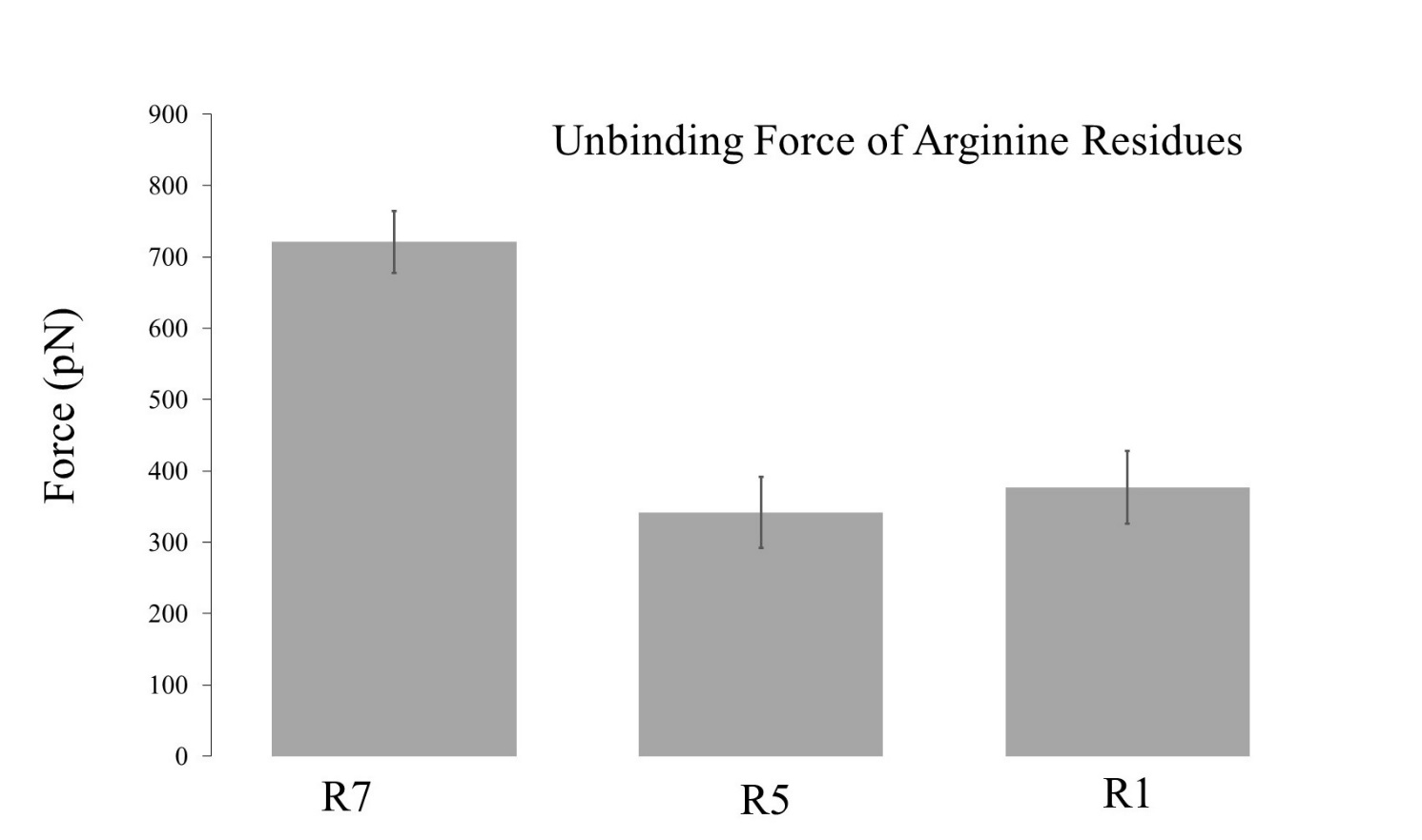


**Figure S2.** Unbinding force of arginine residues in 10 repeats of MD simulations.


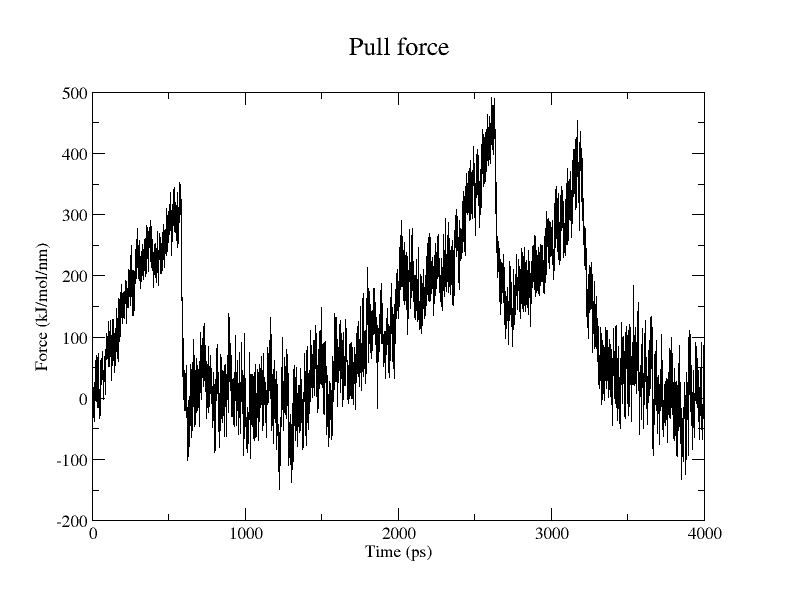


**Figure S3**. Force-time traces of pulling on N-terminal of SB7 from silica surface in MD simulations.


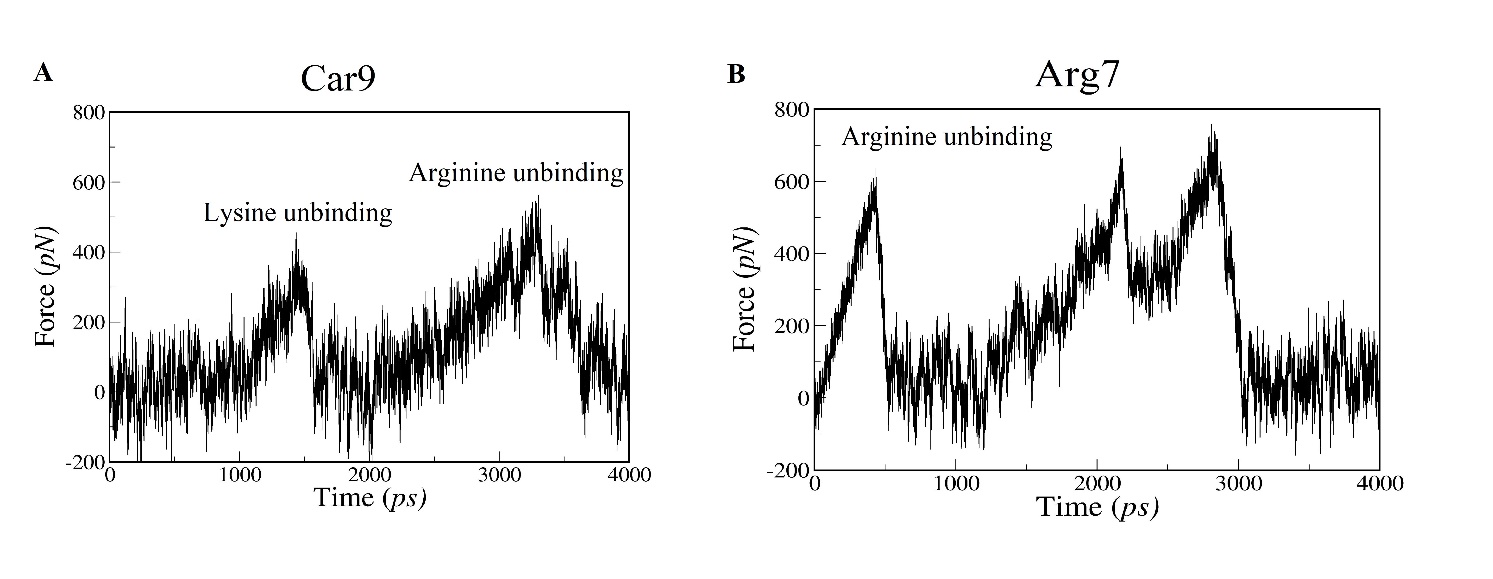


**Figure S4**. Force-time traces of pulling Car9 (A) and Arg (B) from the silica surface in MD simulations.


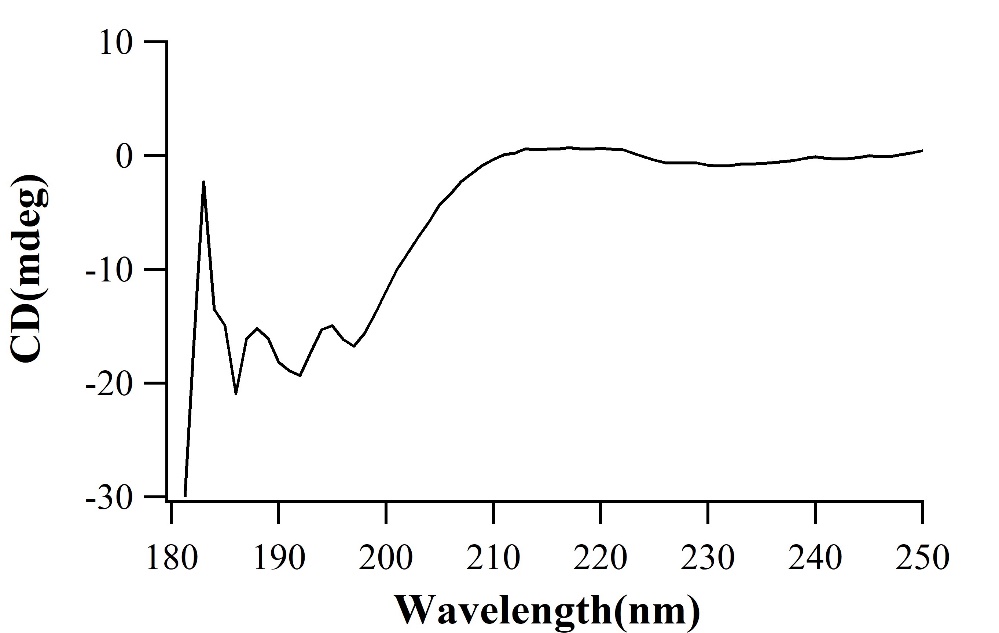


**Figure S5.** Circular dichroism spectroscopy on SB7. The minima around 195 nm suggests that SB7 has a random coil conformation.
